# Comparing biosignatures in aged basalt glass from North Pond, Mid-Atlantic Ridge and the Louisville Seamount Trail, off New Zealand

**DOI:** 10.1371/journal.pone.0190053

**Published:** 2018-02-21

**Authors:** Andreas Türke, Bénédicte Ménez, Wolfgang Bach

**Affiliations:** 1 Department of Geosciences and MARUM, University of Bremen, Klagenfurter Str. GEO, Bremen, Germany; 2 Institut de Physique du Globe de Paris, Sorbonne Paris Cité, Univ Paris Diderot, CNRS, France; 3 Centre of Excellence in Geobiology and the Department of Earth Sciences, Realfagbygget, University of Bergen, Allegaten 41, Bergen, Norway; Medical University Graz, AUSTRIA

## Abstract

Microbial life can leave various traces (or biosignatures) in rocks, including biotic alteration textures, biominerals, enrichments of certain elements, organic molecules, or remnants of DNA. In basalt glass from the ocean floor, microbial alteration textures as well as chemical and isotopic biosignatures have been used to trace microbial activity. However, little is known about the relationship between the physical and chemical nature of the habitat and the prevalent types of biosignatures. Here, we report and compare strongly variable biosignatures from two different oceanic study sites. We analyzed rock samples for their textural biosignatures and associated organic molecules. The biosignatures from the 8 Ma North Pond Region, which represents young, well-oxygenated, and hydrologically active crust, are characterized by little textural diversity. The organic matter associated with those textures shows evidence for the occurrence of remnants of complex biomolecules like proteins. Comparably the biosignatures from the older Louisville Seamount Trail (~70 Ma) are more texturally diverse, but associated with organic molecules that are more degraded. The Louisville Seamount has less fresh glass left and decreased permeability, which metabolic pathways may dominate that only leave molecular biosignatures without textural evidence of glass alteration. We propose that diverse biosignatures in oceanic crust may form during different stages of crustal evolution.

## Introduction

Subseafloor basalts are one of the largest and most intriguing habitats on Earth. Oceans cover two-thirds of Earth’s surface and the upper 500 m of the underlying igneous crust represents Earth’s largest aquifer [1].

Evidence for microbial life in the basaltic ocean crust has been found in a number of different areas and settings (e.g. in modern ocean crust [2;3] or in Archean rocks from greenstone belts [4;5]) and by different lines of evidence (referred to as biosignatures from here on). The preservation of biosignatures has been shown to be dependent on the degree of alteration and remineralization during diagenesis and fluid/rock interaction in terrestial systems [6]. Initial work had used textural criteria to highlight microbial activity in subseafloor basalts, namely putative ichnofossils, preserved as granular and tubular alteration textures in basalt glass [3;7;8;9], which come in different shapes and sizes [10]. They are usually between 1–5 μm in width and tens to hundreds of μm in length and are hypothesized to be formed by etching of the volcanic glass as a result of microbial activities. It has been suggested that these microbes thrive on organic matter or oxidize Fe^2+^ and Mn^2+^ from the glass for chemolithoautotrophic energy gain with electron acceptors like O_2_, sulfates, or CO_2_ from seawater [7]. The origin of alteration textures is best explained by microbially mediated dissolution of glass [11], as their spatial distribution and relationship to one another, as well as their geometry contradict suggestions of abiotic origin [12]. Furthermore, sulfur isotope values in sulfide minerals [13], phylogenetic studies [14;15], attest for the presence of active microbial communities within the basaltic ocean crust, whose abundance and diversity has been studied by molecular work. Microorganisms have been shown to enhance alteration rates of volcanic glass [16;17]. The abiotic alteration of basalt glass is called palagonitization and is a very sluggish process, in which fresh glass is replaced mainly by clay minerals and zeolites. Therefore microbes seem to play an important role in determining the rate of exchange between oceanic crust and seawater.

Yet, the extent and role of the marine subseafloor basalt hosted biosphere remains largely unexplored, as drilling campaigns into the upper oceanic crust have suffered from low recovery of ~ 25% [18]. Drilling tends to collect more massive rock lithologies which are likely less suitable for microbial colonization. Porous and more altered rocks on the other hand are samples less often, but are preferable as a habitat, as fractures facilitate essential fluid transport. Furthermore contamination of samples has been a major issue for microbiological studies of the basalt hosted subseafloor biosphere [19]. Those difficulties are mainly combated with the installation of Circulation Obviation Retrofit Kits (CORK) [20;21] after drilling and coring into the cased borehole. CORKs allow for monitoring and sampling of basement fluids, and incubation experiments with predefined mineral substrates to study the redox chemistry of the subseafloor habitat. Studies at the Juan de Fuca Ridge utilizing CORKs have revealed an active and diverse microbial basement community [22] that also shifted as redox conditions in the borehole changed [23;24]. However, observatories are limited to a few subseafloor environments and have only been employed for roughly two decades. Very little is known about the relationship between subseafloor microbial activity and the chemical and physical evolution of the subseafloor habitat through time [18;25;26]. The chemical reactions available for chemolithoautotrophic energy gain in this habitat are strongly influenced by the crustal evolution and its chemical change during seafloor weathering and advancing sedimentation as the basaltic crust ages [27;28]. Microbial colonization of seafloor basalts has been shown to occur within seafloor samples as young as less than 20 ka [14]. As for prokaryotes, there is evidence that fungal communities colonize basalts relatively early, as they are fossilized within secondary minerals like zeolite and calcite, which precipitate as veins in cracks or in vesicles close to the ridge [29;30].

But do communities, and consequently biosignatures change with ongoing alteration of basaltic ocean crust in seafloor weathering, which affects the bioenergetical and physical conditions of this subseafloor environment? And, furthermore, which microorganisms form which kind of biosignatures?

Here we report differences in tubular biosignatures and associated organic matter in samples from the 8 Ma North Pond area, near the Mid-Atlantic Ridge and the Louisville seamounts, northeast of New Zealand (up to ~80 Ma), which are two distinctly different sites in terms of their crustal evolution. Age of ocean crust plays a crucial role, as it dictates factors such as the crust hydrological state, the extent of fluid-rock interactions, the abundance and type of secondary minerals, and the magnitude of geochemical change. These different factors influence the microbial communities and their potential in altering ocean crust along with the associated biosignatures.

## Material and methods

### Analytical strategy

We sampled basalt glass from drill cores with a focus on areas of secondary mineral formation (alteration fronts). Furthermore, we thoroughly documented the types of tubular alteration textures in the volcanic glass and assessed their spatial relationship to alteration fronts. We then used Scanning Electron Microscopy (SEM) and Fourier Transform InfraRed (FTIR) microscopy to locate and characterize the organic matter associated with basalt glass alteration.

### Study sites

We analyzed glassy rock samples from drill cores U1372A and U1376A from Integrated Ocean Drilling Program (IODP) Expedition 330 [31] to the Louisville Seamount Trail and from drill cores U1382A and U1383C from IODP Expedition 336 [32] to North Pond on the western flank of the Mid-Atlantic Ridge (22°45’N and 46°05’W). The drilling campaigns were carried out in 2010 and 2011 by IODP in international water and did not require an access permit. No protected species were sampled for the research presented.

The Louisville Seamount trail [33] is a 75 km wide volcanic chain of guyots and seamounts in the South Pacific. The basement age at Site U1372 is 74 Ma, whereas it is 64 Ma at Site U1376 [31]. The entire igneous section of the drill cores has undergone low-temperature alteration. Oxygen isotope compositions of abundant carbonate veins and breccia cement indicate alteration temperatures below 20°C in both holes [34]. Negative Ce-anomalies of the carbonates from Hole U1372A may suggest oxidizing conditions. In contrast, carbonates from Hole U1376A lack a Ce anomaly but show weak positive Eu anomalies, indicative of stronger basement influence and less oxidizing conditions [34].

The North Pond region is a sediment pond of 8×15 km in size, located on the western flank of the Mid-Atlantic Ridge in 8 Ma basement. The sediment pond of up to ~200 m thickness is surrounded by steep rift mountains that act as recharge and discharge zones of circulating seawater. Heat flow data indicate rapid SE-NW directed movement of large amounts of seawater at temperatures of 2–15°C [35]. This open circulation of oxygenated water within the North Pond basaltic basement leads to abundant oxygen in the aquifer fluids in the entire North Pond area [36;37]. Basement alteration is dominated by conversion of glassy flow margins and hyaloclastites to palagonite and zeolite [28] and still ongoing.

Drilling derived contamination was assessed with the use of fluorescent microspheres of 0.52 μm diameter were added to the drill fluid as a particulate tracers mimicing microbial cells at both sites. While microsphere were present in the drill cores of both IODP expeditions [31;32], we did not observe any microspheres in the samples analyzed in this study.

### Sample preparation

Two types of samples were analyzed for the work presented here:

1. A set of 18 thin sections of 30 μm thickness (11 from Louisville and 7 from North Pond), each one of which has > 100 individual tubular alteration textures. These thin sections were not used in the search for organic molecules due to contamination issues in the thin section preparation involving organic resin and glue.
2. Pieces of the same samples were prepared for the identification of organic molecules. Samples were cleared from external parts and then cut into thin slices, so that all surfaces were fresh cuts. We used a saw blade cleaned with 5% sodium hypochlorite and rinsed with sterilized ultrapure water. Afterwards the slices were polished by hand on silicon carbon abrasive discs with absolute ethanol to a thickness of approximately 100 μm and analyzed via scanning electron microscopy and Fourier Transformation Infrared microscopy. At all stages the rocks were only handled with clean sterile nitrile gloves. This procedure should limit contamination with organic and post sampling colonization.

### Microtunnel count and description

For 18 of 27 total thin sections of 30μm thickness, we characterized 100 randomly chosen individual microtunnels under a transmission light microscope according to Fisk & McLoughlin’s Atlas of alteration textures in volcanic glass [10]. The textures were classified based on their size (long ≥ 20μm in length; short < 20μm in length; thin < 2μm in diameter; thick > = 2μm in diameter), curvature, variations in width #1 (constant; tapered), variations in width #2 (none; engorged; bumpy; annulated; mushroom), branching (none; simple, mossy, network, palmate, crown), and content (empty; dark contents; ovoid bodies; internal division; filament; patterned; septae).

### Scanning electron microscopy

We used a Zeiss Supra 55 VP Field Emission® SEM coupled to energy dispersive X-ray spectrometry (EDS) at the Université Pierre et Marie Curie (Paris, France). Images were collected with a beam acceleration voltage of 15 kV using a backscattered electron (BSE) detector (AsB). EDS analyses and chemical mapping were carried out with a Bruker Xflash Quad® spectrometer using the microanalysis system Quantax. All the samples analyzed by SEM were prepared free of epoxy resin to avoid organic contamination (see “Sample preparation” section) and Au-coated. The samples were treated in an ultrasonic bath to remove particulates from all surfaces.

### Fourier transformation infrared microscopy

FTIR measurements were carried out at the Institut de Physique du Globe de Paris, France with a Thermo Scientific iN10 MX® (Ever-Glo infrared source) equipped with a ×15 objective (NA = 0.7) and a liquid nitrogen cooled MCT-A detector in transmission and reflection mode. We acquired spectra in the range 4000–710 cm^-1^ by mapping over areas of several mm^2^ with individual spot sizes of 30×30 μm and steps of 30 μm. Each spectrum corresponded to 128 summed scans collected with a spectral resolution of 4 cm^-1^. The raw data was converted into–ASCII files with the Omnic Atlus® imaging software by Thermo Scientific^TM^ and afterwards normalized and background-reduced using the ChemoSpec package for the software R [38].

The vibrational bands summarized in Table 1 were considered as indicative of bonds or functional groups in organic molecules, which potentially originate from biological material [39;40].

**Table 1:** Vibrational band wavenumbers, assignment and potential molecular origin in biological material, as compiled by Movasaghi et al. [39] and Preston et al. [40].

Wavenumber (cm^-1^)	Assignment	Potential origin in biological material
2956	CH_3_ asymmetrical stretch	Lipids
2920	CH_2_ asymmetrical stretch	Lipids
2870	CH_3_ symmetrical stretch	Lipids
2850	CH_2_ symmetrical stretch	Lipids
1743	C = O stretch	Esters
1715	C = O stretch	Carbonic acids
1680–1715	C = O stretch	Nucleic acids
1653	Amide I, C = O stretch, C-N stretch, N-H bend	Proteins
1567–1548	Amide II, N-H bend, C-N stretch	Proteins
1470	CH_3_ asymmetrical bend	Lipids, Proteins
1468	CH_2_ scissoring	Lipids
1460	CH_3_ asymmetrical bend	Lipids, Proteins
1430	C-O-H in-plane bending	Carboxylic group
1401	N^+^ (CH_3_)_3_ symmetrical bend	Lipids
1320	C-O stretch	Carboxylic group
1299	Amide III, C-N stretch, N-H bend, C = O stretch, O = C-N bend	Proteins

### R_3/2_ value

The R_3/2_ value was first introduced by Lin and Ritz [41] and Marshall et al. [42] in order to characterize biopolymer aliphaticity composition and elucidate the biological origin of microfossils, based on FTIR spectroscopy. It is the CH_3_/CH_2_ absorbance ratio of the respective asymmetric stretching band heights. The R_3/2_ value hence reflects the degree of branching and chain length of side groups associated with aliphatic chains. Low R_3/2_ values suggest that mainly long or less-branched aliphatic carbon chains as one would expect for bacterial or eukaryotic membrane phospholipids. Conversely, archaeal lipids lack true fatty acid side chains and hence yield higher R_3/2_ values. Igisu et al. [43] recently showed that depending on their phylogenetic affiliation at the domain level, cells yield contrasted R_3/2_ values (Eukarya approximately 0.3–0.5; Bacteria 0.6–0.7; Archaea 0.8–1.0). Individual bacterial cell components however, plot significantly higher (proteins) or slightly (membranes) to significantly (lipids) lower compared to whole cell R_3/2_ values [43]. We used the R_3/2_ value to compare the fossilized organic matter found within the glassy basalts from the North Pond region and the Louisville Seamounts and unravel their connection to tubular alteration textures.

## Results

### Tubular alteration textures

**Fig 1:**
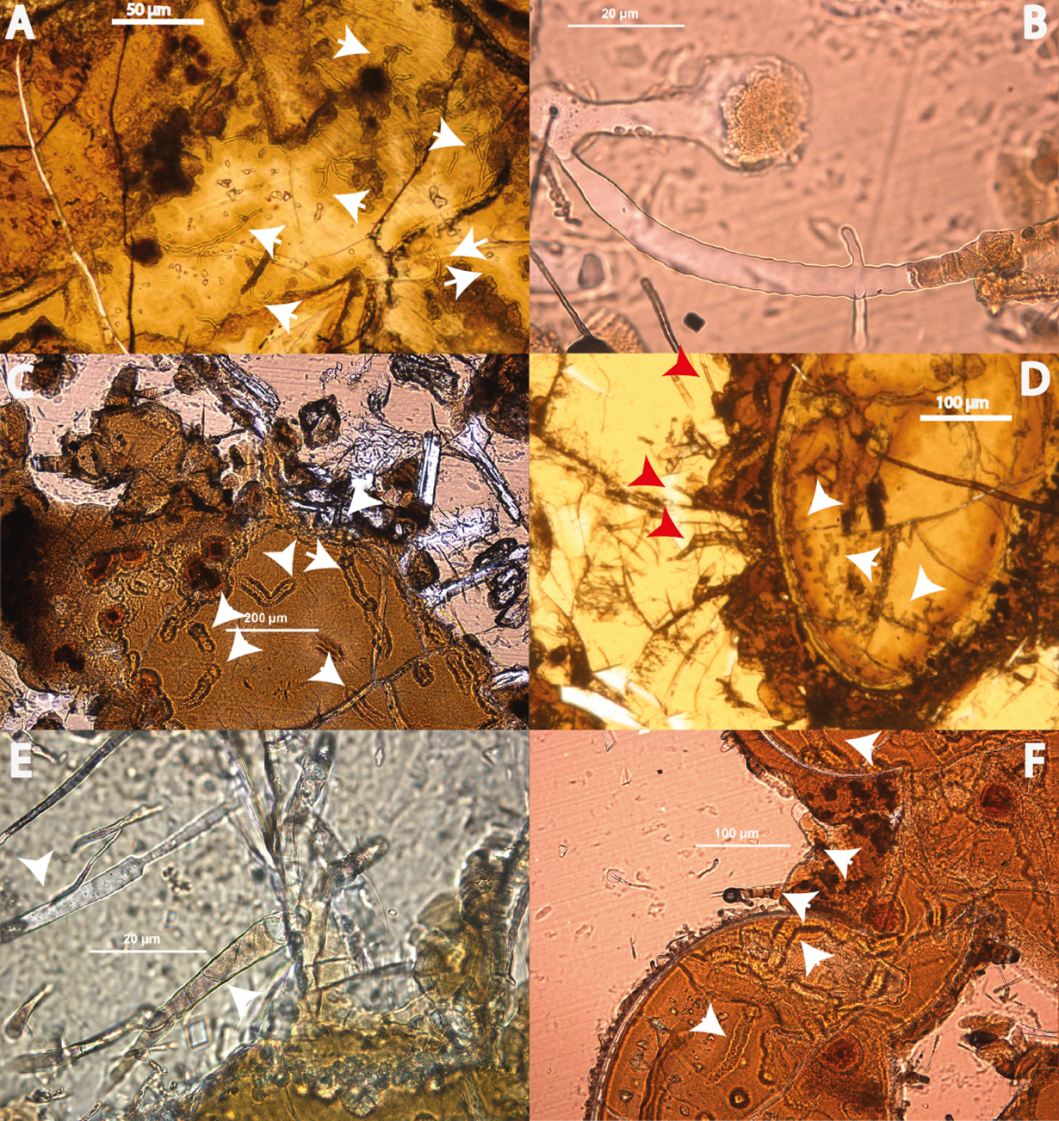
Some of the remarkable tubular alteration textures in the Louisville samples in volcanic glass under plane polarized light. A (U1376A-16R-6W-65/71): Long, thin, and branched tubular alteration textures preserved in palagonite and indicated by white arrows. B (U1376A-16R-6W-65/71): A single long, wide, and branched tubular alteration texture preserved in glass. C (U1376A-16R-6W-65/71): Several very large tubular alteration textures preserved in palagonite and indicated by white arrows. D (U1372A-19R-1W-79/81): Remnants of tubules preserved (or formed) in palagonite (white arrows), red arrows show them protruding into fresh glass. E (U1372A-29R-3W-77/80): Long and wide tubule, which shows significant variation in width at the terminus along with simple branching F (U1376A-16R-6W-65/71): Significantly large textures preserved in palagonite, similar to those shown in D. The length of the scale bars is 50 μm in A, 20 μm in B, 200 μm in C, 100 μm in D, 20 μm in E, and 100 μm in F.

**Fig 2:**
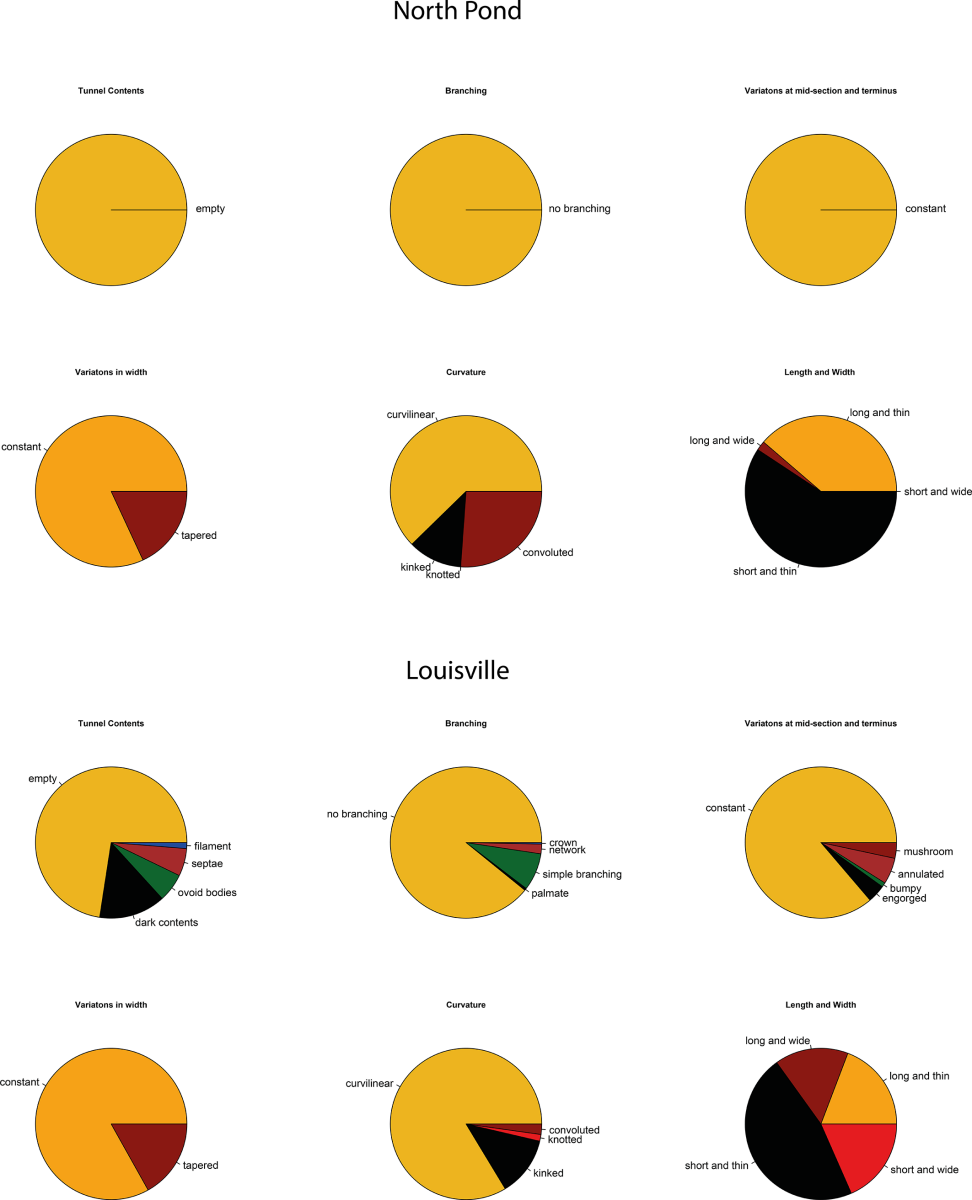
Types of tubular alteration textures found in the North Pond and Louisville samples. In Louisville basalt glass, short and thin tubes make up almost 50% of the samples, but longer and thicker tubes are prevalent as well, compared to the North Pond samples. Furthermore, Louisville samples are more diverse in terms of variations in widths, branching, and tunnel contents, compared to the North Pond samples. For North Pond basaltic glass, thin tubes whether long or short, make the majority of the samples, and thick tubes are almost completely absent. They do not show any branching, are exclusively empty, and do not show any variations in width.

The tubular alteration textures as identified by transmitted light microscopy (Fig 1) were classified following [10]. Most of the texture types described were found within the samples from Louisville, whereas the diversity of textures was lower in the North Pond samples (Fig 2).

The tubules (N = 700, with N the number of examined textures) in the North Pond samples were almost exclusively < 2 μm in width (98%) and there were more short tubes (59%), than long tubes (41%). Curvature was wide spread, with 62% of the tubes being curvilinear, 12% being kinked, and 26% convoluted. There were no knotted tubes identified in the North Pond samples. Variation in width was very low, the majority being uniform in width (82%); only 18% of the tubes were tapered, and there was no variation at mid-section and terminus, nor any branching or tunnel contents.

Conversely, the Louisville tubular alteration textures (N = 1100) on the other hand were much more diverse in every respect. Short and thin tubules were the most abundant ones (47%), similar to North Pond, however long and thin (19%), short and wide (18%), and long and wide tubes (16%) were commonly found as well throughout all the samples. The variation in curvature was less pronounced, but all types were present, with 84% being curvilinear, 13% kinked, 1% knotted, and 2% convoluted. Variations in width were very common. Although most of the tubes were uniform in width, similarly to the North Pond textures (83% constant, and 17% tapered), there was some variations at mid-section and terminus, with 4% engorged, 1% bumpy, 5% annulated, and 3% mushroom textured. Unlike in the North Pond samples, branching occurred in the Louisville tubes, although 88% of the tubes were not branched. 8% showed simple branching, whereas complex branching was less prominent (network 2%, palmate, mossy, and crown < 1%). Furthermore only 73% of the tubules were empty, 14% were filled with dark contents, 6% with ovoid bodies, 6% showed septae, and 1% were filled with filaments. Ovoid bodies and septae were exclusively found in long and wide tubules.

### Organic compounds

We found the presence organic compounds both in samples from the North Pond area and the Louisville Seamounts. However, they were distinctly different in their location and composition.

**Fig 3:**
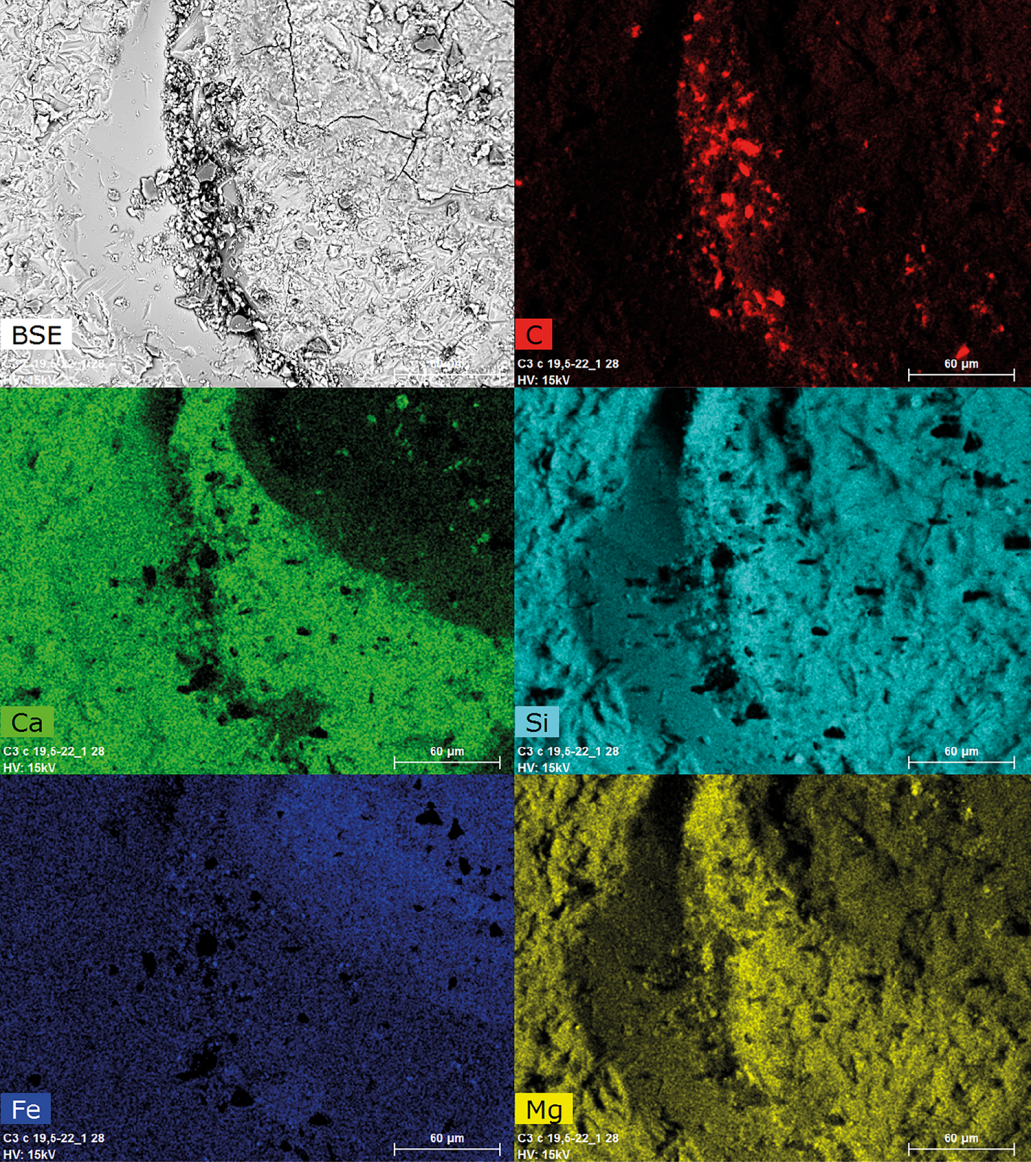
North Pond sample U1383C-29R-1W-6/9: SEM image (backscattered electrons (BSE)) and associated EDS elemental maps for C, Ca, Si, Fe, and Mg collected at 15 kV accelerating voltage. Carbon coming from the organic matter, appearing as dark spots of less than ten micrometers on the BSE image, is located closely to the Fe-enriched palagonite/Ca-enriched glass interface. They are particularly concentrated in the glass hollow from which palagonite was removed.

**Fig 4:**
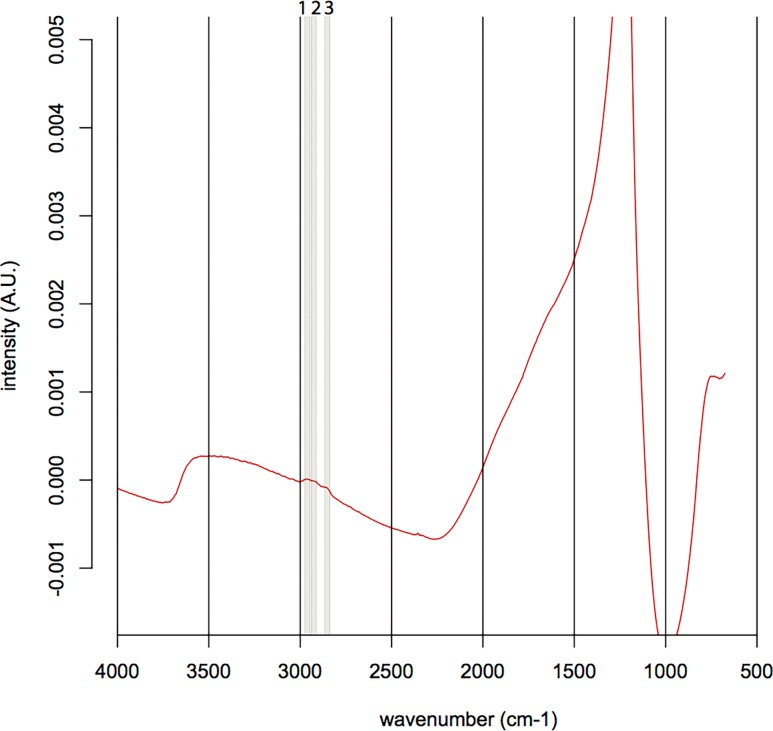
Typical FTIR spectrum of the organic matter found in the North Pond sample U1383C-29R-1W-6/9. The presence of organics is suggested by the following absorption bands: 1: CH_3_ asymmetrical stretch, 2: CH_2_ asymmetrical stretch, 3: CH_2_ symmetrical stretch, 4: C = O stretch, 5: Amide I band, 6: Amide II band, 7: CH_3_ and CH_2_ scissoring and asymmetric bends, 8: Amide III band (Table 1). The broad main around 1250 cm^-1^ absorption band corresponds to the Si-O bond. A.U. stands for arbitrary unit.

The North Pond samples contained only little carbon-rich phases, which appeared as tiny aggregates exclusively restricted to the palagonite/glass interface (Fig 3). SEM-EDS analysis revealed that carbon was not present as carbonate, nor as SiC contamination from polishing, as it was not associated with Ca, Mg, Fe, or Si enrichments. The FTIR spectra of the organic matter found in the North Pond samples (Fig 4) showed indications for the presence of remnant lipids (aliphatic absorption bands at 2956 cm^-1^, 2920cm^-1^, 2850 cm^-1^, and 1460–1470 cm^-1^ representing the asymmetrical stretch of CH_3_, the asymmetrical stretch of CH_2_, the symmetrical stretch of CH_2_, along with the asymmetrical bend of CH_2_/CH_3_, and the scissoring of CH_2_, respectively). Furthermore, the spectra showed evidence for the presence of esters/fatty acids and/or polysaccharides (absorption band at 1743 cm^-1^ representing the C = O stretching mode) and proteins (absorption bands at a 1653 cm^-1^, 1550 cm^-1^, and 1300 cm^-1^ representing Amide I, II, and III, respectively along with the asymmetric bend of CH_2_/CH_3_ between 1460–1470 cm^-1^). Associated R_3/2_ values ranged from 0.45 to 1.20.

**Fig 5:**
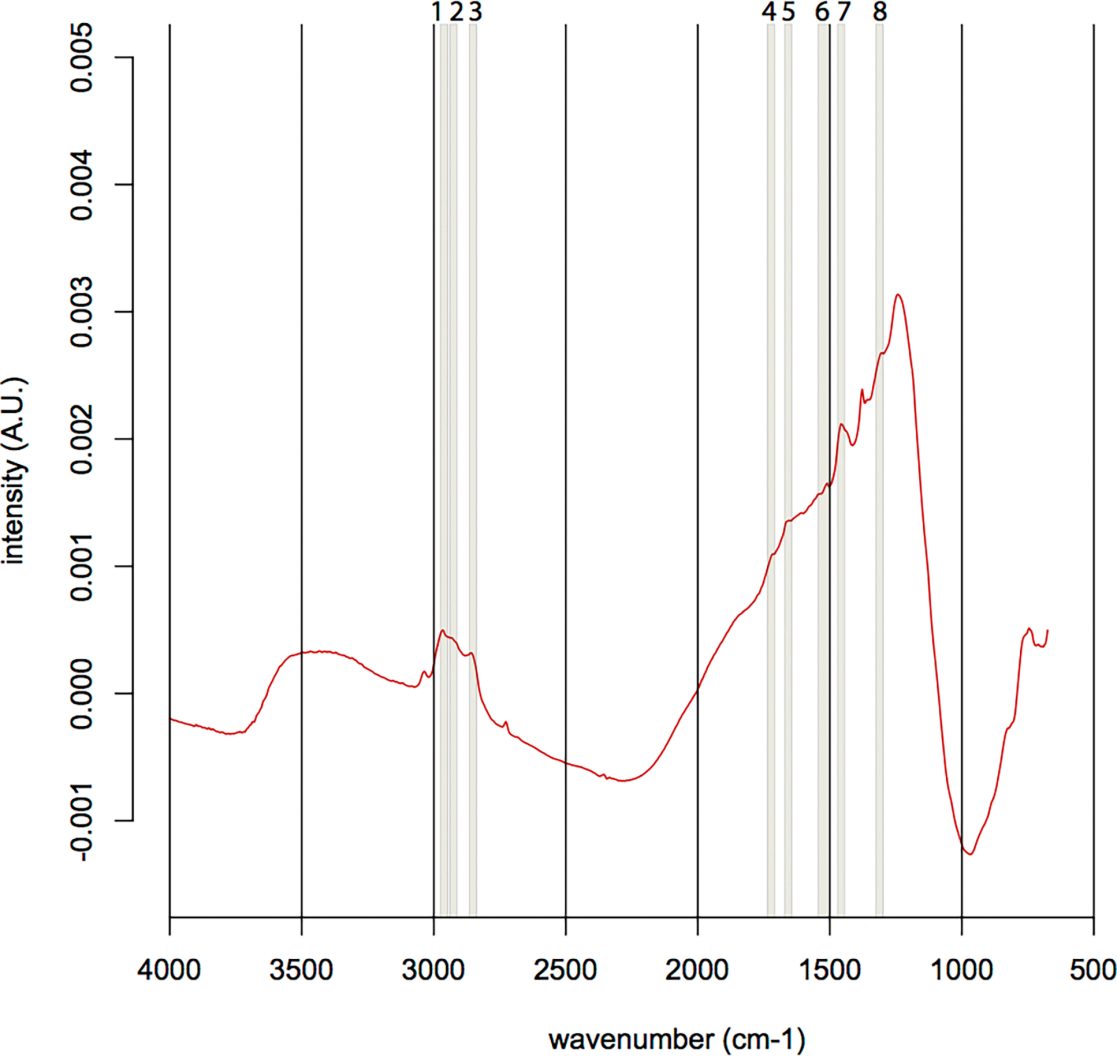
Typical FTIR spectrum of the organic matter found in the Louisville sample U1376A-16R-6W-65/71. The absorption bands indicate the presence of organics. 1: is the CH_3_ asymmetrical stretch, 2: is the CH_2_ asymmetrical stretch, and 3: the CH_2_ symmetrical stretch. The broad main around 1250 cm^-1^ absorption band corresponds to the Si-O bond. A.U. stands for arbitrary unit.

**Fig 6:**
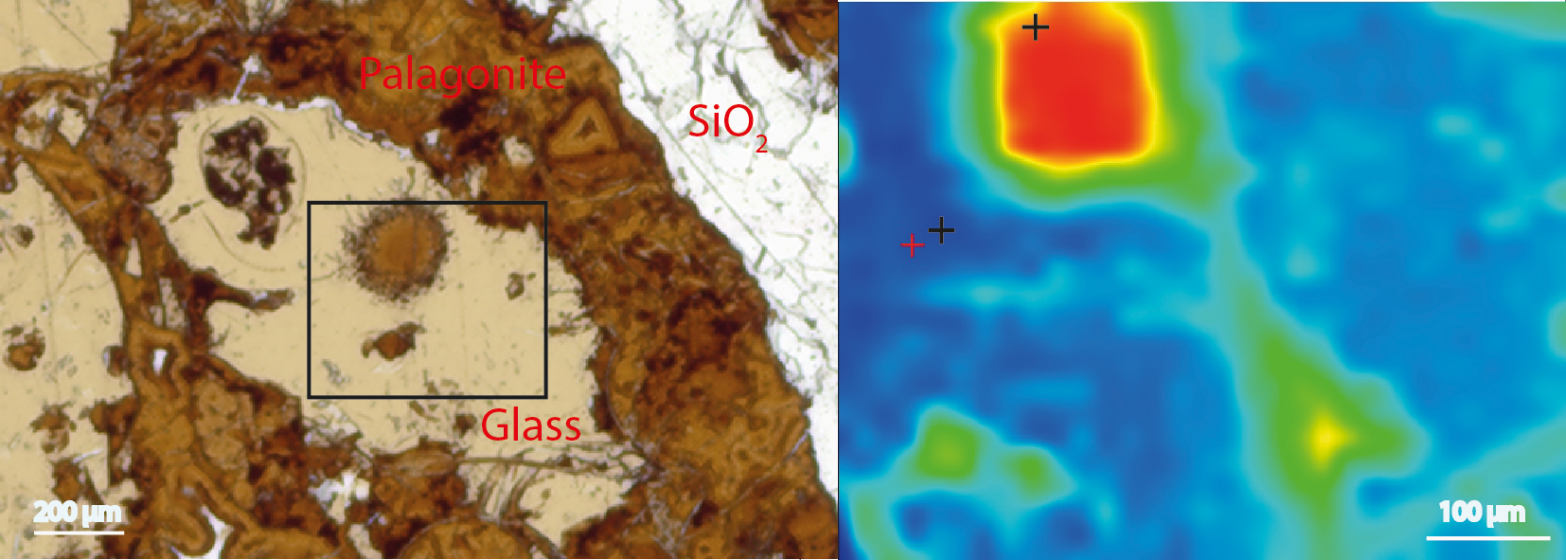
(A) “negative” split of a basalt glass from Louisville sample U1376A-16R-6W-65/71 (resin-free thin section), as shown under plane polarized light; (B) associated FTIR map highlighting the spatial distribution of the CH_3_ vibrational band. The FTIR map location is shown by the black rectangle in (A) and indicates highest abundance of organic matter in the palagonite-filled vesicle, from which tubular alteration textures protrude into the surrounding volcanic glass. The scale bars are 200 μm (left) and 100 μm (right).

**Fig 7:**
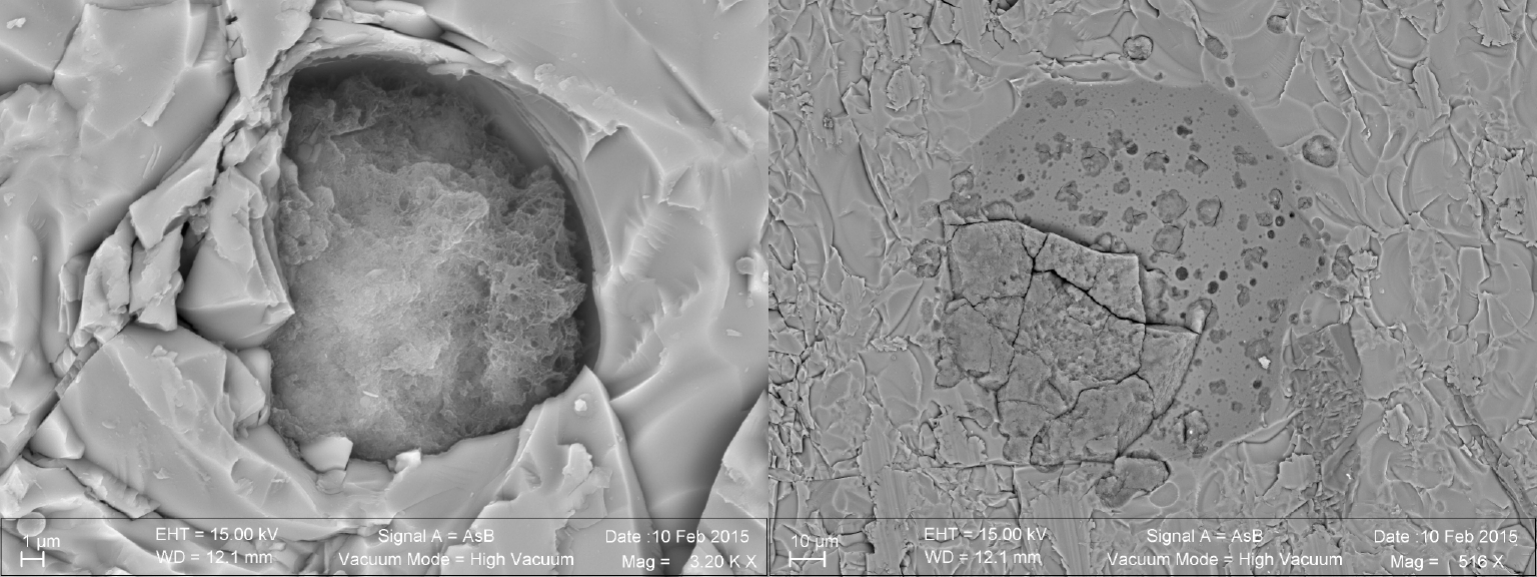
SEM images in BSE mode of a vesicle in a basalt glass (light grey) filled with palagonite (dark grey) (Louisville sample U1376A-16R-6W-65/71). Several etch pits are visible on the vesicle surface on the right; the image on the left shows a close up view of an etch pit, which protrudes into the volcanic glass as the onset of a tubular alteration texture.

On the opposite, as shown by the FTIR spectrum in Fig 5, the samples from Louisville contained less complex organic matter, which appeared to consist exclusively of aliphatic chains that may be attributed to lipids (vibrational bands at 2956 cm^-1^, 2920cm^-1^, and 2850 cm^-1^ representing the asymmetrical stretch of CH_3_ and CH_2_ as well as the symmetrical stretch of CH_2_, respectively). These long chain hydrocarbons were widely spread in the samples and occurred both in palagonites and vein filling zeolites or SiO_2_ (Fig 6). However, compared to the North Pond, small C-rich aggregates (as shown in Fig 3) were never associated with the numerous many etch pits that were observed in the fresh glass (Fig 7), and were identified as the onset of tubular alteration textures. In addition, associated R_3/2_ values were much less scattered and ranged from 0.78 to 1.01.

## Discussion

### Differences in biosignatures

**Fig 8:**
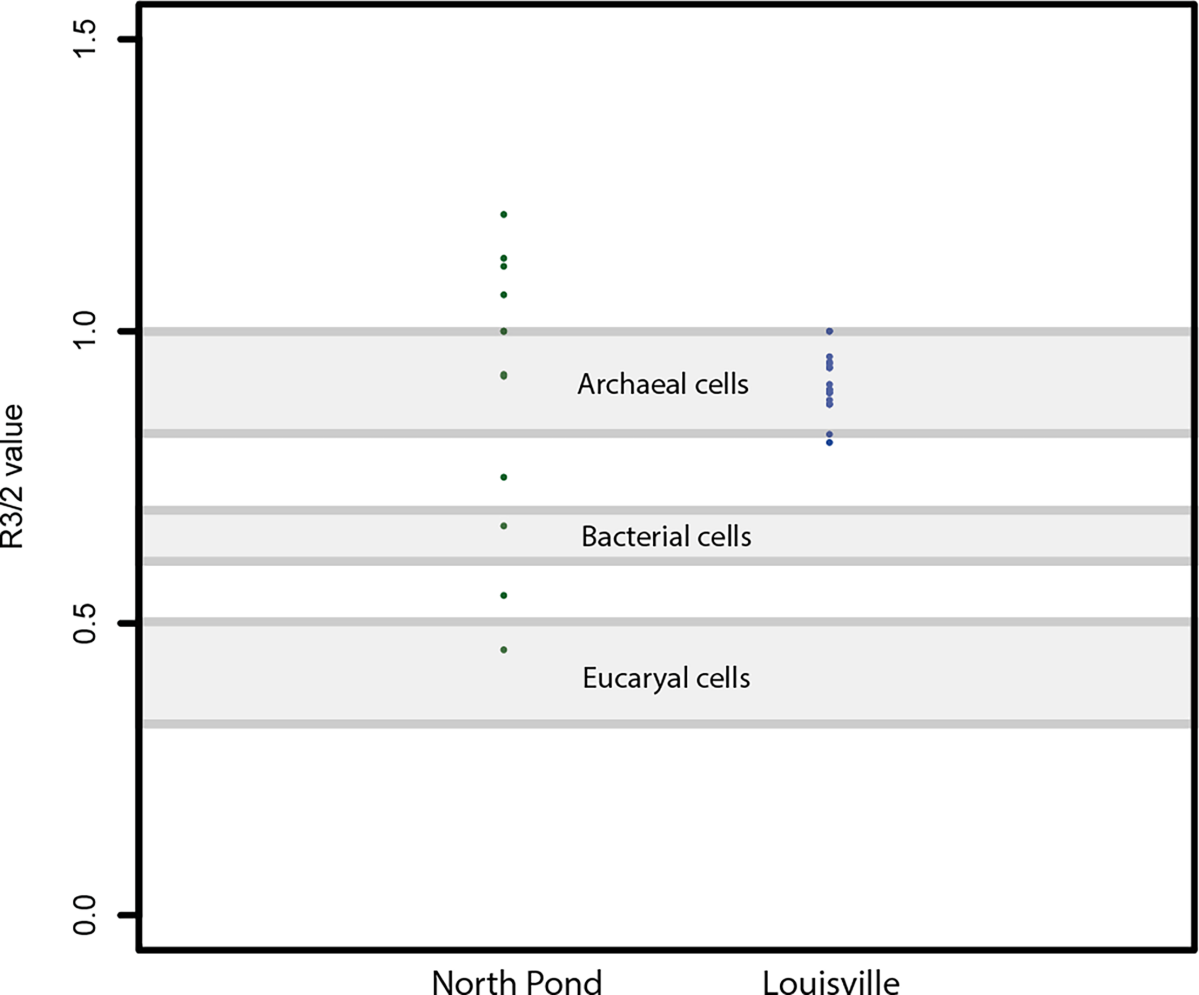
R_3/2_ values as deducted from FTIR analysis of the organic matter found in the North Pond and Louisville samples. The values mostly plot above the values of whole bacterial and eukaryotic cells in both cases [43;44]. Note that, individual cell constituents like membranes and lipids have lower R_3/2_ values than whole cells, whereas proteins plot higher.

We argue that the differences in the biosignatures of North Pond and Louisville are influenced by their different age, geological history, and the influence of both parameters on the basaltic aquifer as a microbial habitat. North Pond represents a rather young (8 Ma) area of ridge flank, which has undergone and is still undergoing oxidative alteration with temperatures at or below 20°C. Here, tubular alteration textures are rare, thin, and show little morphological diversity, with only small variations in width, curvature, and branching (Fig 2). The low abundance and diversity might be related to the young age of the crust, which allowed only few and simple alteration textures to form. The organic matter we were able to detect in the North Pond samples via FTIR microscopy was sparse and showed, among others indications, remnants of proteins (Fig 4). As proteins are expected to degrade quickly, their presence in the samples may indicate that the biological material is rather fresh. Their strict location at the fresh glass/palagonite interface (Fig 3), where tubular alteration initiates, potentially suggests adhering colonies taking advantage of the altering rock releasing energy sources and nutrients. Based on Igisu et al. [43;44], no clear conclusions can be drawn from the R_3/2_ values regarding the microbial community structure, as they span over a very wide range (0.45–1.20; Fig 8). However, the highest R_32_ values fall within and even above the range of archaeal cells, whose membrane lipids constituents are made of isoprenoid chains that are highly branched and subsequently have higher R_3/2_ values compared to bacteria. High R_3/2_ values are also consistent with the presence of proteins remnants among the detected pool of organic components, since proteins have R_3/2_ values that are increased relative to other cellular components [44]. As FTIR spot sizes of 30 μm exceeded the size of individual cells, these values likely represent mixed analyses of various types of microbes.

**Fig 9:**
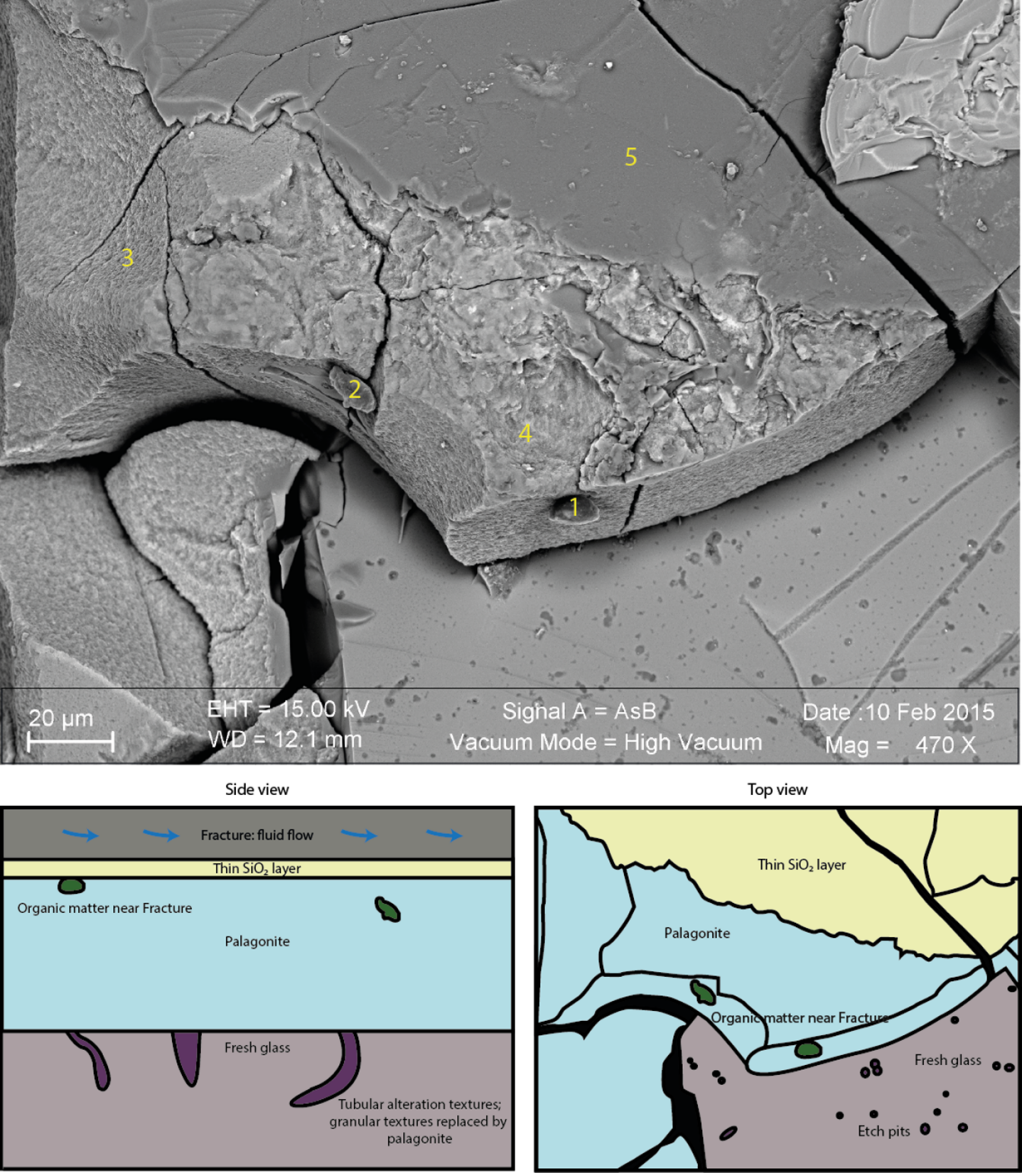
top: SEM image of Louisville sample U1376A-15R-3W-37/41 in BSE mode showing organic matter microaggregates (1 & 2) within a palagonite rim (3 & 4) on top of fresh glass with several etch pits and below an SiO_2_ filled fracture (5). bottom: sketch as side view (bottom left) and top view (bottom right), illustrating the spatial distribution of the tubular biosignatures and the organic matter remnants. The tubes originate from the fracture/fresh glass interface and propagate into the glass, are then overgrown by palagonite and sealed by precipitating SiO_2_. The organic matter was only found within palagonites, suggesting that these putative remnants of microbial life were not associated with formation of tubular alteration textures.

The Louisville samples are much older (~70 Ma [33]) and have undergone a longer, but less well constrained history of alteration, although borehole U1376A was shown to be influenced by basement, hence suggesting less oxidizing conditions for those samples. Here, the tubular biosignatures are more diverse and complex (Fig 2). The much larger tubular features preserved in palagonites (Fig 1C and 1F) resemble trace fossils of fungal communities found in other oceanic basalts [29]. However, the preserved organic matter was more degraded compared to North Pond and only yielded FTIR absorption bands of aliphatic compounds (Fig 5) with R_3/2_ values in the range of the ones predicted from archaeal lipids [43]; Fig 8) Rather than being located close to the surface of fresh glass as for the young North Pond samples, the organic matter was detected in secondary void fills such as palagonite in former vesicles (Fig 6) or vein fills with SiO_2_ (Fig 9). This indicates that the organic matter was either flushed into the aquifer and then fossilized during mineral precipitation within fractures and vesicles, or that the organic matter is indigenous to the rock, and was primarily associated with void spaces where microbial communities likely thrive attached on the rock.

The relationship between alteration (and thus mineral/fluid interface) and biosignatures is further illustrated in Fig 9. Tubular alteration textures in basalt glass form early, when the glass is exposed to oxygenated seawater (and hence oxidants) in fractures and oxidation of iron and sulfur can supply energy for microbial life. The tubular alteration textures can be partially replaced later by palagonitization byproducts, which act as a barrier and inhibits supply of energy via oxidation of glass [28]. Eventually tertiary minerals (like zeolites, carbonates or SiO_2_) seal fractures and fluid flow ceases. When basalt ages, the organic matter is found within the palagonites and close to the sealing SiO_2_, which indicated to us that it is not directly associated with the formation of tubular alteration textures. It could either be added to the rocks from circulating seawater, which may drop dissolved organic carbon in the basement upon water-rock interactions (e.g. [45]), perhaps by formation of solid hydrocarbon/kerogen [46]. The organic matter could also be particulate organic matter that was trapped in the altered rock and then fossilized. Another possibility is that the organic matter is related to later stages of colonization by communities that do not gain their energy from alteration and oxidation of glass. Instead they could possibly use energy sources unrelated to the direct alteration of glass. Türke et al. [28] suggested, that oxidation of radiolytically produced dihydrogen and associated reduction of Fe^3+^ via molecular hydrogen may be the dominant microbial energy sources in old and thoroughly altered basalts. The Louisville basalts reach U concentrations of up to 3 ppm [47], so that significant H_2_ production is expected. This would be in agreement with the observation that organic matter is found (i) near the palagonite/fresh glass interface in young basalts where the glass likely serves as solid energetic substrate (North Pond) and (ii) within palagonites close to fractures in older basalts (Louisville) where hydrogen might be produced by water radiolysis.

Results from molecular ecology studies [48] suggested that the basalts of the Louisville Seamount Chain still host microbial life and anaerobic Mn-oxidizing bacteria have recently been isolated from a basaltic breccia in 341 m depth at site U1374A at Louisville [49]. Additionally, >99% of the prokaryotic community in Louisville subsurface samples were comprised of bacteria [50] and samples from site U1374A were closer in similarity between each other than compared to the U1376A site.

As the R_3/2_ values observed at Louisville contrast the studies of present the present day microbiology at Louisville (not mainly bacterial), we suggest that the biosignatures observed within the subseafloor basalts have not been formed by the present day microbial community and have rather been produced earlier in the crustal evolution. However, our data does not suggest any apparent relation between rock alteration and the presence of organic matter. The two sets of observations may be reconciled either if the rocks presently merely provide a shelter for microbes that derive their metabolic energy from percolating seawater, as proposed by Templeton et al. [51] for altered basalts, or they are rather indicative of degraded fossilized organic molecules from past microorganisms.

In any case, this is an interesting observation as it implies that the community structure of the basalt hosted subseafloor biosphere changes with changing conditions during crustal evolution.

## Conclusion

As the geochemical environment changes significantly during crustal evolution (e.g. oxic in during early stages to suboxic when buried under sediments), basalts might host different communities during different stages of crustal aging.

Our findings support the idea that microbial life in the young basaltic aquifer at North Pond, which alters oxidatively, is energetically driven by glass oxidation processes. The reduced form of iron is found at the glass interface, where the organic matter in the North Pond samples is also preserved. In the much older basaltic crust of Louisville that has undergone a considerably more complex alteration history, the textural biosignatures textures are also more complex and diverse. In the latest stages, life seems to be hosted in voids within the rock, such that basalt acts as a solid support to grow on. The organic matter is then preserved when these voids are filled by secondary phases like calcite, zeolite, or SiO_2_.
